# Microwave-Assisted Synthesis of Zn_2_SnO_4_ Nanostructures for Photodegradation of Rhodamine B under UV and Sunlight

**DOI:** 10.3390/nano12122119

**Published:** 2022-06-20

**Authors:** Ana Rovisco, Maria Morais, Rita Branquinho, Elvira Fortunato, Rodrigo Martins, Pedro Barquinha

**Affiliations:** CENIMAT/i3N, Department of Materials Science, School of Science and Technology, NOVA University Lisbon and CEMOP/UNINOVA, 2829-516 Caparica, Portugal; md.morais@campus.fct.unl.pt (M.M.); ritasba@fct.unl.pt (R.B.); emf@fct.unl.pt (E.F.); rfpm@fct.unl.pt (R.M.)

**Keywords:** Zn_2_SnO_4_, ZTO, polyhedrons, nanoparticles, nanoplates, microwave-assisted hydrothermal synthesis, photocatalysis, rhodamine B

## Abstract

The contamination of water resources by pollutants resulting from human activities represents a major concern nowadays. One promising alternative to solve this problem is the photocatalytic process, which has demonstrated very promising and efficient results. Oxide nanostructures are interesting alternatives for these applications since they present wide band gaps and high surface areas. Among the photocatalytic oxide nanostructures, zinc tin oxide (ZTO) presents itself as an eco-friendly alternative since its composition includes abundant and non-toxic zinc and tin, instead of critical elements. Moreover, ZTO nanostructures have a multiplicity of structures and morphologies possible to be obtained through low-cost solution-based syntheses. In this context, the current work presents an optimization of ZTO nanostructures (polyhedrons, nanoplates, and nanoparticles) obtained by microwave irradiation-assisted hydrothermal synthesis, toward photocatalytic applications. The nanostructures’ photocatalytic activity in the degradation of rhodamine B under both ultraviolet (UV) irradiation and natural sunlight was evaluated. Among the various morphologies, ZTO nanoparticles revealed the best performance, with degradation > 90% being achieved in 60 min under UV irradiation and in 90 min under natural sunlight. The eco-friendly production process and the demonstrated ability of these nanostructures to be used in various water decontamination processes reinforces their sustainability and the role they can play in a circular economy.

## 1. Introduction

Paper, textile, cosmetic, pharmaceutical, and plastics industries use a high amount of organic dyes and compounds, which contribute to contaminating water resources [1]. Among the vast types of contaminants, textile dyes are the most common, and they are not only toxic but also potentially carcinogenic [2,3]. Contaminated water resources can have harmful effects on aqueous ecosystems and thus on human health [1]. The concern regarding the adverse effects of human actions led to the search on how to decompose contaminants, for which photocatalysis is a suitable option. This process was discovered in 1972 and can be applied for water splitting, the degradation of pollutants, whether organic or inorganic, and the removal of heavy metals [4]. Briefly, during the photocatalytic process, the degradation of toxic contaminants into environmentally friendly remains by the photocatalyst occurs when the latter is irradiated with sufficiently energetic radiation [2,3]. When the photocatalyst absorbs ultraviolet (UV), visible, or infrared radiation, the generation of electron –hole pairs occurs on its surface, which is the fundamental process for pollutant degradation [5,6]. Many photocatalysis studies are performed using UV radiation, although visible light represents approximately 50% of the solar spectrum, whereas UV light represents only ≈5%. The extensive use of UV radiation during photocatalytic studies is due to the relatively large optical band gaps of many photocatalytic materials. A high band gap means that only high-energy radiation can stimulate the formation of electron–hole pairs.

Numerous oxide semiconductors present themselves as low-cost and eco-friendly alternatives for the degradation of various pollutants through photocatalysis treatment processes [1]. Semiconductors present several advantages compared to insulator materials, especially concerning the band gap. Semiconductors’ band gap is much lower, which means less energetic radiation is needed to promote the photocatalytic process [7]. Among the semiconductors, various metal oxides have been evaluated as photocatalysts to break down different water pollutants [7,8]. Particularly, metal oxide semiconductor nanostructures are versatile materials that present optical band gaps between UV and visible light energy [9]. Moreover, metal oxide semiconductor nanostructures produced by the solution process typically exhibit higher defect density when compared to the nanostructures obtained through vapor-phase methods, which can boost their photocatalytic activity when irradiated with lower-energy radiation [6,10]. There are several reports of photocatalytic studies performed with metal oxide materials such as titanium oxide, zinc-based oxides, bismuth-based oxides, and manganites [3,8,11,12,13,14,15,16,17,18]. Binary compounds have been proven to have great catalyst properties; however, multicomponent oxides (i.e., with two or more cations) show higher stability in aqueous environments [15]. Additionally, the multicomponent oxide materials’ properties can be tuned by adjusting their cationic ratio.

Among the multicomponent oxide materials, zinc-tin oxide (ZTO) is a low-cost and environmentally friendly material with a wide range of attractive properties. This metal oxide can crystallize in two different phases: Zn_2_SnO_4_ and ZnSnO_3_. Zn_2_SnO_4_ has good stability in adverse conditions, a wide band gap (3.4–4.0 eV) [19], and high mobility already achieved for specific nanostructures (112 cm^2^ V^−1^ s^−1^) [20]. Additionally, Zn_2_SnO_4_ is ZTO’s most stable phase and presents a cubic inverse spinel structure [9,21]. In the inverse spinel structure, the tetrahedral A-sites are occupied by some Zn^2+^ ions, whereas the octahedral B sites are occupied by the other Zn^2+^ and by Sn^4+^ ions. This ion’s inverted order leads to a non-stoichiometric material, resulting in essential properties in regard to catalyst applications, such as high electron mobility [22], which leads to an enhancement in the migration of charge carriers (electron–hole pair) to the catalyst’s surface which increases the rate of the reactions where this catalyst is involved [23]. Another attractive feature of ZTO is the existence of abundant tin and zinc in its composition instead of critical elements, converting this multicomponent oxide into a promising eco-friendly material. The amount of generated waste nowadays points to the need to rely on eco-friendly products and production processes [24].

ZTO nanostructures have been applied in several fields such as energy harvesting [25,26,27,28,29], electronics [20,30,31,32,33], and gas sensing [34,35,36]. Moreover, this metal oxide has been widely studied as a photocatalyst [15,16,21,37,38,39,40,41,42,43]. Most photocatalytic studies performed using ZTO resort to UV irradiation since Zn_2_SnO_4_ presents a band gap in the UV region [38,44,45]. However, a few groups have reported the use of ZTO nanostructures as photocatalysts under visible light irradiation [9,46,47,48]. Table 1 summarizes the photocatalytic activity of different ZTO nanostructures in the degradation of pollutants. Results achieved in the present study for multiple forms of ZTO nanostructures under UV light and sunlight can be found in the tables of Section 3.3 and Section 3.4, respectively.

**Figure 1:**
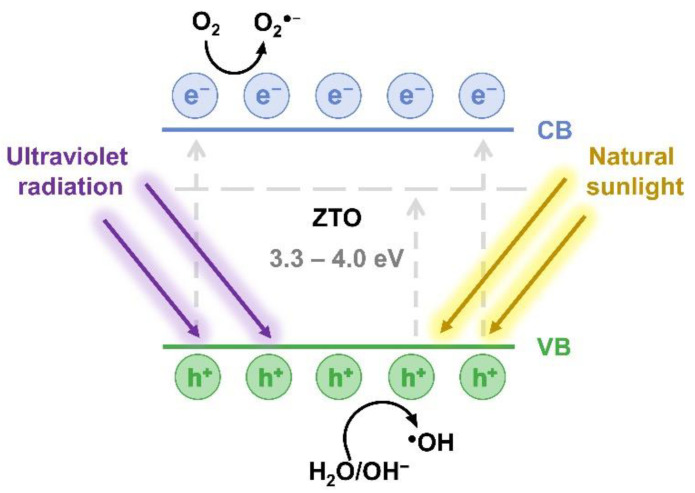
Representative schematic of the photocatalytic mechanism of ZTO nanomaterials.

The mechanism behind the photocatalytic activity of ZTO nanostructures is represented in Figure 1, following the works of refs [2,37,48,51]. Briefly, when ZTO nanostructures are irradiated with radiation with energy above their band gap, i.e., in the UV region, the creation of electron–hole pairs occurs. Visible light irradiation can also promote the creation of these pairs when there is a large density of sub-gap states, arising either due to extrinsic doping or intrinsic defects (e.g., oxygen vacancies) [48]. The electrons excited to the conduction band will then react with the molecular oxygen (O_2_) adsorbed on the semiconductor surface, producing reactive oxygen species such as superoxide anions (O_2_^●−^). The holes present in the valence band react with H_2_O-related groups producing reactive OH species. Both the superoxide anions and the reactive OH species react with the dye molecules promoting their mineralization [52,53].

Various shapes of ZTO nanostructures can be obtained by low-cost hydrothermal methods [54]. Microwave-assisted hydrothermal synthesis has emerged as a time-efficient alternative to the long synthesis time associated with certain conventional hydrothermal syntheses, which require the use of an oven. This methodology is an interesting approach to synthesizing inorganic nanostructures since it presents selective heating and faster reaction rates, derived from the microwave interaction with the chemical species, where dipole rotation and ionic conduction are the two fundamental mechanisms for energy transfer [55]. The decrease in synthesis time and faster heating process contribute to enhancing the product purity and yield [56,57,58,59]. Although scarce, some studies of microwave-assisted synthesis of ZTO nanostructures have already been reported [48,58,60,61]. In this regard, Lehnen et al. reported a simple method to produce quantum dots [58], whereas Nehru et al. performed a urea-based combustion process to produce Zn_2_SnO_4_ nanostructures [60]. More recently, Jain et al. synthesized Zn_2_SnO_4_ nanorods by applying a power of 600 W for 15 min. After the synthesis, the nanostructures underwent annealing treatment at 700 °C for 5 h [48]. Foletto et al. also synthesized Zn_2_SnO_4_ nanoparticles with a power of 1000 W (which corresponds to 250 °C and 60 bar) for several synthesis durations [62].

In the present work, the photocatalytic ability of Zn_2_SnO_4_ nanostructures in the degradation of the common dye RhB under both UV and natural sunlight irradiation was studied. Zn_2_SnO_4_ nanostructures (nanoparticles, nanoplates, and polyhedrons) were synthesized through a low-cost, one-step, seed-layer-free, hydrothermal synthesis assisted by microwave irradiation at only 200 °C. The Zn_2_SnO_4_ nanoparticles (10 min of synthesis duration) revealed the best photocatalytic performance, leading to RhB degradation of >90% in 60 min under UV irradiation and >93% in only 90 min under natural sunlight exposure. These nanostructures were tested with reactive oxygen species scavengers to understand the fundamental entities involved in the photocatalytic degradation of RhB. Moreover, these nanoparticles were reused, showing their potential to be used in multiple water decontamination treatments. To the best of our knowledge, this is the first work to report a comparison of the photocatalytic efficiency of Zn_2_SnO_4_ nanoparticles, nanoplates, and polyhedrons synthesized by microwave hydrothermal synthesis under natural sunlight and UV irradiation.

## 2. Materials and Methods

### 2.1. Synthesis of Zn_2_SnO_4_ Nanostructures

Zn_2_SnO_4_ nanoplates and polyhedrons were synthesized via a hydrothermal method, based on the process presented in [63], but replacing the conventional oven with a microwave system (based on the preliminary findings reported in [64]). Zinc chloride (ZnCl_2_, ACS, CAS: 7646-85-7 from Merck, Darmstadt, Germany), tin (IV) chloride pentahydrate (SnCl_4_·5H_2_O, 98%, CAS: 10025-69-1 from Riedel de Haën, Honeywell, Charlotte, NC, USA), and sodium hydroxide (NaOH, ≥98%, CAS: 1310-73-2 from Sigma-Aldrich, Lisbon, Portugal) were used without further purification. In a typical synthesis, 0.04 M of zinc chloride and 0.02 M of tin (IV) chloride were dissolved separately in 7.5 mL of deionized water. Afterward, the two were mixed, and the mineralizer sodium hydroxide was added (0.24 M). The solution was then transferred to a 35 mL Pyrex vessel, which was then placed in a CEM Discovery SP microwave (from CEM, Matthews, NC, USA). The synthesis was carried out at 200 °C in the dynamic mode using a maximum power of 80 W and a maximum pressure of 270 PSI. Aiming to obtain nanoplates and polyhedrons, four synthesis times were explored (15, 30, 45, and 60 min).

Zn_2_SnO_4_ nanoparticles were also synthesized through a microwave-assisted hydrothermal approach. For the synthesis, 0.34 g zinc chloride and 0.44 g tin (IV) chloride were dissolved separately in 5 mL deionized water and then mixed. Then, 0.53 g sodium carbonate (Na_2_CO_3_, 99.9%, CAS: 497-19-8 from Merck, Darmstadt, Germany) was dissolved in 5 mL deionized water and added dropwise to the precursors’ mixture under magnetic stirring, and the resulting solution was allowed to react for 15 min [65]. The obtained solution was transferred to a 35 mL Pyrex vessel, which was then placed in a CEM Discovery SP microwave (from CEM, Matthews, NC, USA). The synthesis was carried out at 200 °C in the dynamic mode under a maximum power of 100 W and a maximum pressure of 270 PSI. Several synthesis times were studied (5, 10, and 20 min).

After the synthesis, the resulting precipitates were washed several times with deionized water and isopropanol and centrifuged at 6000 rpm for 5 min (high-speed Neya 8, Remi, Maharashtra, India). Then, the nanostructures were dried in a vacuum at 60 °C for 2 h.

### 2.2. Structural, Morphological, and Optical Characterization of Zn_2_SnO_4_ Nanostructures

The crystalline structure of the Zn_2_SnO_4_ nanostructures was analyzed by X-ray diffraction (XRD) using a PANalytical’s X’Pert PRO MRD diffractometer (PANalytical B.V., Almelo, The Netherlands), with a monochromatic Cu Ka radiation source. Data were acquired in a range between 10° and 90° (2θ) with a scanning step size of 0.016°.

The morphology of the Zn_2_SnO_4_ nanostructures was evaluated by scanning electron microscopy (SEM) using a Carl Zeiss AURIGA CrossBeam (FIB-SEM) workstation equipped with an Oxford X-Max 150 X-ray Energy Dispersive Spectrometer (Carl Zeiss Microscopy GmbH, Oberkochen, Germany).

Reflectance measurements were carried out at room temperature using a PerkinElmer Lambda 950 UV/VIS/NIR spectrophotometer (PerkinElmer, Waltham, MA, USA) with a diffuse reflectance module with a 150-mm-diameter integrating sphere, internally coated with Spectralon. The system’s calibration was performed using a Spectralon reflector sample as reference (R = 1.0). The reflectance spectra were acquired from 350 to 800 nm.

### 2.3. Evaluation of the Zn_2_SnO_4_ Nanostructures Photocatalytic Performance

The nanostructures were tested as photocatalysts under both UV radiation and natural sunlight. First, the photocatalytic performance of the Zn_2_SnO_4_ nanostructures under UV radiation was evaluated by the degradation of RhB (C_28_H_31_ClN_2_O_3_, ≥95%, CAS: 81-88-9 from Sigma-Aldrich, Lisbon, Portugal) at room temperature. For each experiment, 40 mg of each sample was dispersed in 50 mL of the RhB aqueous solution (5 mg/L). The solutions with Zn_2_SnO_4_ nanostructures were first stirred for 30 min in the dark to establish the adsorption–desorption equilibrium and then exposed to UV radiation using three mercury lamps, model HNSL from Osram Puritec (Osram, HNS L 95 W 2G11, Munich, Germany), each with a power of 95 W and an emission wavelength range between 200 and 280 nm (ozone free). The solutions were aligned in parallel at a distance of 27 cm from the UV lamps. The absorption spectra of the RhB solution with the dispersed nanostructures were recorded at intervals of 15 min until a total of 60 min UV exposure was reached. The photocatalysts’ performance was also tested under natural sunlight around midday for a total exposure of 90 min. Sunlight intensity was measured to be around 98 mW/cm^2^ (0.98 sun) during the experiments by a solar power meter from Sciencetech (Sciencetech-Inc., London, ON, Canada). During these experiments, the absorption spectra of the RhB aqueous solutions with the dispersed nanostructures were recorded at intervals of 15 min, during the first 30 min, and then with intervals of 30 min until a total of 90 min sunlight exposure was reached.

The absorption spectra were recorded using a PerkinElmer Lambda 950 UV/VIS/NIR spectrophotometer (PerkinElmer, Waltham, MA, USA) in the wavelength range between 250 and 800 nm. For each absorption measurement, 4 mL of the RhB solution with the dispersed photocatalysts was collected and centrifuged. After the absorbance measurements, the RhB solution and the nanostructures were returned to the container for further UV or sunlight exposition.

For the reusability assays, after each photocatalytic experiment, the catalysts were washed several times alternatively with deionized water and isopropanol. The powders were collected by centrifugation at 6000 rpm for 5 min (high-speed Neya 8, Remi, Maharashtra, India). Afterward, the nanostructures were dried in a vacuum, at 60 °C for 2 h, before each reusability trial. For this study, additional parallel photocatalytic experiments were performed to ensure that the second and third cycles were performed using the same Zn_2_SnO_4_ mass as in the first cycle (40 mg).

### 2.4. Investigation of the Reactive Oxygen Species (ROS) Involved in the Photocatalytic Degradation of RhB by Zn_2_SnO_4_ Nanostructures

Various reactive oxygen species (ROS) scavengers were used to investigate the species and the mechanisms involved in the photocatalytic degradation of RhB by Zn_2_SnO_4_ nanostructures when irradiated with either UV radiation or natural sunlight. In these studies, ethylenediaminetetraacetic acid (EDTA, C_10_H_14_N_2_Na_2_O_8_·2H_2_O, ≥99%, CAS: 6381-92-6 from Scharlau, Barcelona, Spain) was used as a hole (h^+^) scavenger, hydrogen peroxide (H_2_O_2_, >30% *w/v*, CAS: 7722-84-1 from PanReac AppliChem, Darmstadt, Germany) as an electron scavenger (e^−^), tert-butyl alcohol anhydrous (TBA, C_4_H_10_O, ≥99.5%, CAS: 75-65-0 from Sigma-Aldrich, Lisbon, Portugal) as a hydroxyl radical (•OH) scavenger, benzoquinone (BQ, C_6_H_4_O_2_, Pharmaceutical Secondary Standard, CAS: 106-51-4 from Sigma-Aldrich, Lisbon, Portugal) as the superoxide radical (•O^2−^) scavenger, and sodium azide (NaN_3_, NaN_3_, BioXtra, CAS: 26628-22-8 from Sigma-Aldrich, Lisbon, Portugal) as a singlet oxygen (^1^O_2_) scavenger.

The procedure used during the tests with the ROS scavengers to investigate the photocatalytic performance of the Zn_2_SnO_4_ nanostructures was the same either when irradiated with UV radiation or natural sunlight. For these tests, 2.5 mL of each scavenger solution with a molar concentration of 0.5 mM was added to 25 mL of the dye solution with 20 mg of Zn_2_SnO_4_ nanostructures. Since, for convenience, both the catalyst’s mass and dye volume were reduced the exposition time was reduced from 90 min to 30 and 45 min under UV light and natural sunlight irradiation, respectively.

## 3. Results and Discussion

### 3.1. Synthesis of Zn_2_SnO_4_ Nanostructures

Zn_2_SnO_4_ nanostructures with different morphologies (namely nanoplates, polyhedrons, and nanoparticles) were synthesized by two different microwave-assisted hydrothermal methods.

#### 3.1.1. Zn_2_SnO_4_ Polyhedrons and Nanoplates

Zn_2_SnO_4_ polyhedrons were synthesized by a microwave-assisted hydrothermal approach based on previous work, where the synthesis was performed at 200 °C for 24 h in a conventional oven [63]. A posteriori, it was found that Zn_2_SnO_4_ nanoplates were produced by reducing the synthesis duration to 12 h, although this reduction did not allow for obtaining a pure phase [23].

**Figure 2:**
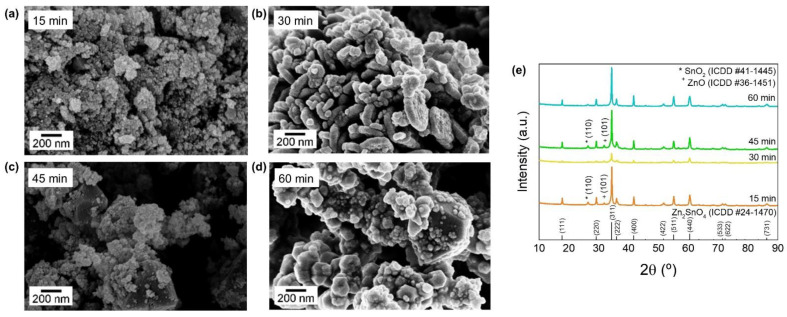
(**a**–**d**) SEM images and (**e**) XRD pattern of the synthesized ZTO nanostructures in the microwave system for 15, 30 (Zn_2_SnO_4_ nanoplates), 45, and 60 min (Zn_2_SnO_4_ polyhedrons).

Following the results presented in [63], herein, the use of a conventional oven as the heating source was replaced by a microwave system to synthesize Zn_2_SnO_4_ polyhedrons and nanoplates. Aiming to achieve these two distinct Zn_2_SnO_4_ morphologies, four synthesis durations were considered: 15, 30, 45, and 60 min. In Figure 2, the SEM images and the XRD patterns of the resultant nanostructures are presented.

Figure 2a–d shows the evolution of the nanostructures’ morphology with the increase in the microwave-assisted hydrothermal synthesis duration. As expected, a similar trend is observed in the nanostructures obtained, whether by microwave or conventional hydrothermal synthesis. For the 30 min synthesis time, the formation of nanoplates can be observed, although a small mixture (a few nanoparticles) can be detected. A 60 min synthesis duration leads to the formation of polyhedrons, as presented in Figure 2d. Meanwhile, the 15 and 45 min syntheses lead to the formation of a mixture of nanostructures with various morphologies. XRD analysis (Figure 2e) confirms that all nanostructures have the Zn_2_SnO_4_ phase (ICDD card 024-1470), since excluding the peak at 26.661°, which is characteristic of SnO_2_ nanostructures (ICDD card 041-1445), and the peak at 31.770° typical of ZnO nanostructures (ICDD card 036-1451), the remaining peaks presented in the diffractograms can be associated with those of Zn_2_SnO_4_ nanostructures. Interestingly, although a mixture of nanostructures is observed for 15 and 45 min, no significant differences are observed in the XRD diffractograms.

The successful synthesis of Zn_2_SnO_4_ nanoplates and polyhedrons by microwave-assisted synthesis in less than 1 h allows the reduction of the synthesis time up to 23 h when compared to conventional oven heating.

#### 3.1.2. Zn_2_SnO_4_ Nanoparticles

The influence of the synthesis time in obtaining Zn_2_SnO_4_ nanoparticles was also studied. Based on previous results, where Zn_2_SnO_4_ nanoparticles were obtained through the conventional hydrothermal technique, the synthesis procedure was adapted to a microwave reactor with the aim of decreasing the synthesis duration. The synthesis was performed in dynamic mode at 200 °C, establishing a maximum power of 100 W and a maximum pressure of 270 PSI. Three synthesis durations were considered: 5, 10, and 20 min.

**Figure 3:**
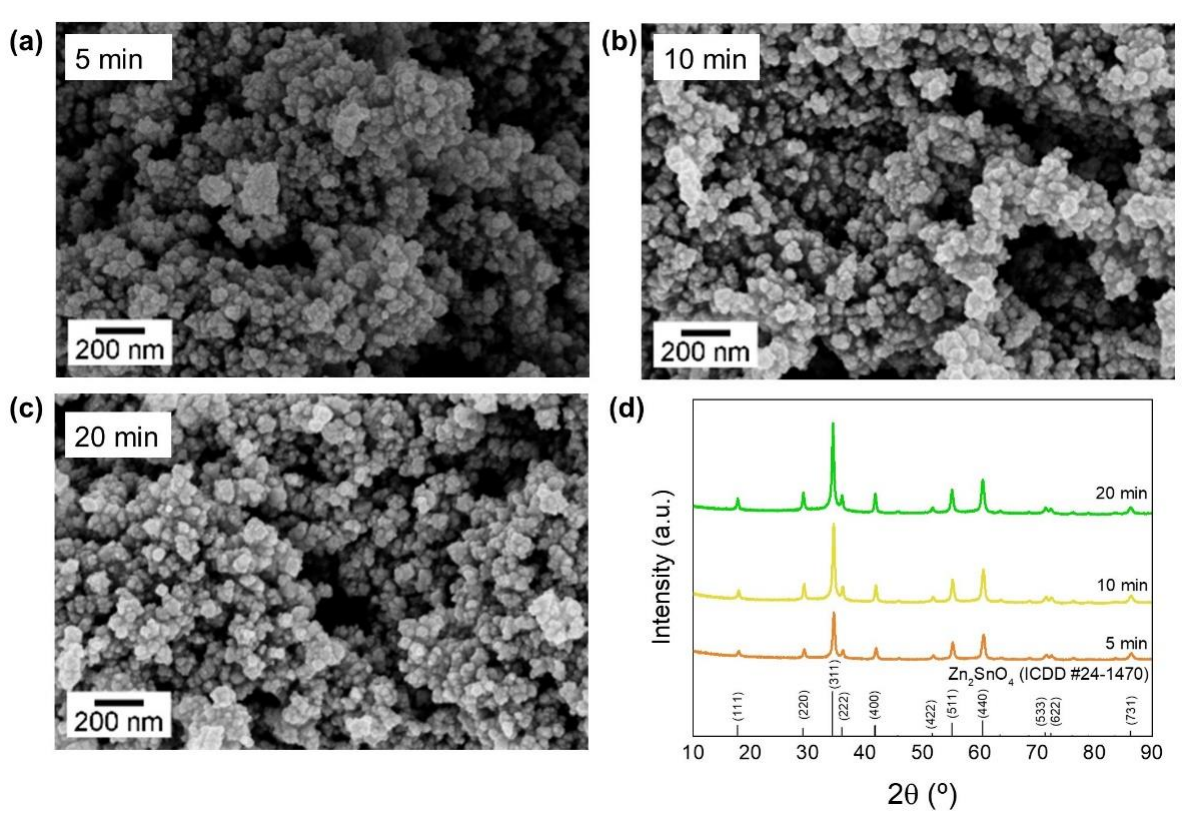
(**a**–**c**) SEM images and (**d**) XRD pattern of the Zn_2_SnO_4_ nanoparticles synthesized by microwave-assisted synthesis for 5, 10 and 20 min.

The SEM images and the XRD patterns of the obtained nanostructures are presented in Figure 3 showing that small nanoparticles with a pure Zn_2_SnO_4_ phase (ICDD card 024-1470) were obtained. This observation indicates that it is possible to reduce the synthesis duration by almost 12 h without compromising the obtention of Zn_2_SnO_4_ nanoparticles with good quality when compared with what was achieved by Annamalai et al. [65]. Annamalai’s group performed a similar hydrothermal method as the one reported in this work but used a conventional oven instead of a microwave reactor. Their results showed an impure Zn_2_SnO_4_ phase after 6 h of synthesis, requiring 12 h to achieve a pure Zn_2_SnO_4_ phase.

In general, the obtained results demonstrate the efficiency of the microwave heating method in the production of high-quality Zn_2_SnO_4_ nanoplates, polyhedrons, and nanoparticles at 200 °C without post-processing annealing treatment, with the advantage of allowing for significantly shorter synthesis times than the ones usually needed when using conventional ovens, namely 60 min for polyhedrons, 30 min for nanoplates, and 5 min for nanoparticles.

### 3.2. Optical Properties of Zn_2_SnO_4_ Nanostructures

The optical band gap (E_g_) of the ZTO nanostructures was estimated through the application of the Kubelka-Munk (K-M) method to the reflectance data [66]. This method is based on the K-M function (F(R)), which can be defined as follows:

(1) $$F\left(R\right)=\frac{{\left(1-R\right)}^{2}}{2R}$$

The K-M function is proportional to the absorption coefficient (α), so by considering the Tauc relation, it is possible to obtain the following expressions [52]:

(2) $$F\left(R\right)\propto \;\alpha \;\propto =\frac{{\left(h\upsilon -E_{g}\right)}^{1/n}}{h\upsilon }$$

(3) $${\left(F\left(R\right)h\upsilon \right)}^{n}=A\left(h\upsilon -E_{g}\right)$$

where A is a constant, hυ is the photon energy, and n takes the value of 2 for the case of semiconductors with direct allowed transitions [67].

The E_g_ of the ZTO nanostructures can be determined by extrapolating the linear part of the K-M curve, presented in Figure S1, with the energy axis. The determined optical band gap values for the Zn_2_SnO_4_ nanoparticles, polyhedrons, and nanoplates are summarized in Table 2, where the average dimensions of the ZTO nanostructures are also presented. The wider band gap of the Zn_2_SnO_4_ nanoparticles, when compared to Zn_2_SnO_4_ polyhedrons and nanoplates, can be attributed to their smaller sizes [65]. In general, with the exception of Zn_2_SnO_4_ nanoparticles obtained through the synthesis using the conventional oven, the increase in the nanostructures’ dimensions leads to a decrease in their optical band gap, as expected.

The optical band gap values of the synthesized ZTO nanostructures show that their optical absorption is in the UV region, which enhances their application as catalysts under UV light irradiation. Regardless, in previous work, the presence of energy levels within the band gap (associated with oxygen vacancies) was detected through photoluminescence (PL) analysis [63]. These levels (represented in Figure 1) exhibit energies in the visible range and thus promote the absorption of photons with energies lower than the ZTO nanostructures’ band gap, allowing for their use as visible light catalysts.

Through the Urbach energy (E_U_), it is possible to compare the defect density of the nanostructured materials. This energy can be determined by the following equations, where α is the absorption coefficient and hν is the photon energy [68]:

(4) $$\alpha \;={\;\alpha }_{0}\;\mathrm{eh}\nu /E_{U}$$

(5) $$\ln\;\alpha \;={\;\ln\;\alpha }_{0}+\left(h\nu /E_{U}\right)$$

The Urbach energies associated with each synthesized Zn_2_SnO_4_ nanostructures were estimated and are presented in Table 2. Regarding the nanoparticles, it can be seen that the increase in the synthesis time leads to a rise in the E_U_, which points to a higher number of sub-gap levels of the nanoparticles obtained in a 20 min microwave-assisted hydrothermal synthesis. Zn_2_SnO_4_ polyhedrons show the lowest defect density, presumably because this is the most stable phase and shape of crystalline ZTO [45,69,70]. These results are in agreement with what was observed in a previous study with the same type of Zn_2_SnO_4_ nanostructures but produced by a conventional hydrothermal synthesis [19]. The observed higher defect density of the Zn_2_SnO_4_ nanoparticles suggests the possible better performance of these nanostructures in the photodegradation of organic dyes under visible light and, consequently, under natural sunlight, since these defects expand the absorption edge towards the visible region [15].

### 3.3. Photocatalytic Activity of Zn_2_SnO_4_ Nanostructures under UV Light

**Figure 4:**
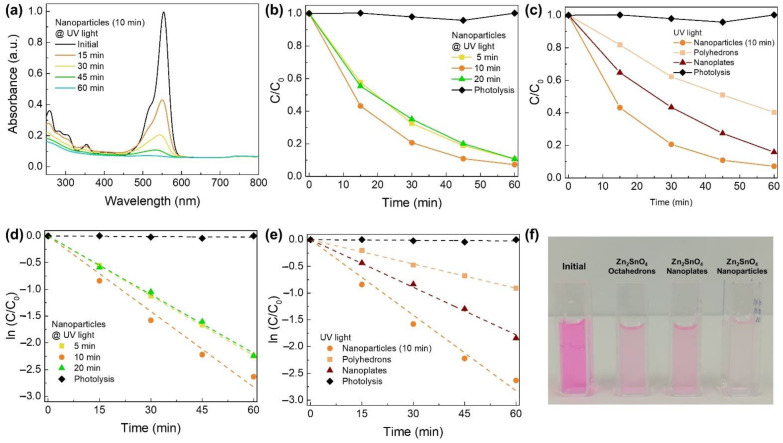
(**a**) Absorbance spectra of the RhB photocatalytic degradation under UV light using as photocatalyst Zn_2_SnO_4_ nanoparticles (10 min synthesis time). C/C_0_ comparison of the RhB photocatalytic degradation (**b**) between ZTO nanoparticles (5, 10, and 20 min synthesis time) and (**c**) between ZTO nanostructures (nanoparticles (10 min synthesis), nanoplates, and polyhedrons). Lines in (**b**,**c**) are for eye guiding only. Kinetic parameters of the RhB degradation under UV light of (**d**) Zn_2_SnO_4_ nanoparticles (5, 10, and 20 min synthesis time), and (**e**) Zn_2_SnO_4_ nanostructures (nanoparticles (10 min synthesis), nanoplates, and polyhedrons). Lines in (**d**,**e**) represent the linear fittings applied for the determination of the degradation rates. (**f**) Photograph images of the RhB solution before and after its degradation for 60 min under UV light using each Zn_2_SnO_4_ nanostructure as photocatalyst. Error bars are not shown as they are not noticeable in the plot due to the low uncertainty values.

The photocatalytic activity of the Zn_2_SnO_4_ nanoplates, polyhedrons, and nanoparticles produced by microwave-assisted hydrothermal synthesis was studied through the degradation of RhB under UV irradiation. Figure 4a and Figure S2 show the absorbance spectra of the RhB solution recorded at different degradation times under UV irradiation in the presence of each ZTO nanostructure. The ratio between the maximum value of each absorbance spectrum (C) at each exposure time and the initial absorbance of the RhB solution (C_0_, mg·L^−1^) can be estimated based on the absorbance spectra presented in Figure 4a and Figure S2. The obtained C/C_0_ values can then be used to plot the kinetic parameters of the RhB degradation associated with each experiment. The C/C_0_ and kinetic parameter graphs are presented in Figure 4.

In order to compare the photocatalytic performance of the different nanostructures, the degradation rate (k) was determined through the pseudo-first-order reaction kinetic model, represented by ln(C_0_/C) = kt [48]. The obtained values are presented in Table 3.

Through the analysis of the various graphs presented in Figure 4 and the values in Table 3, it can be concluded that the nanoparticles exhibit a much higher degradation rate (0.0471 min^−1^) than the polyhedrons (0.0151 min^−1^) and the nanoplates (0.0297 min^−1^). This higher photocatalytic performance can be due to the higher surface area of the nanoparticles (0D) compared with that of the other two nanostructures (2D and 3D), which also can explain the lower degradation rate of the polyhedrons compared with the nanoplates. Regarding the Zn_2_SnO_4_ nanoparticles, the increase in their dimensions leads to a rise in the corresponding degradation rate, which suggests that, in fact, the surface area is a key parameter in the performance of the catalysts.

Interestingly, the nanoparticles synthesized by the microwave-assisted method revealed a much better performance when compared with the ones fabricated in a conventional oven and even when compared with other nanostructures such as nanowires [19]. These results prove that the presented microwave-assisted synthesis is not only a much faster synthesis method but also leads to Zn_2_SnO_4_ nanostructures efficient for water treatment.

In conclusion, among the various morphologies, the highest degradation rates were obtained for the nanoparticles. Between the various synthesis times, a 10 min duration led to obtaining the nanoparticles with the best photocatalytic performance, with RhB degradation >90% in just 60 min.

**Figure 5:**
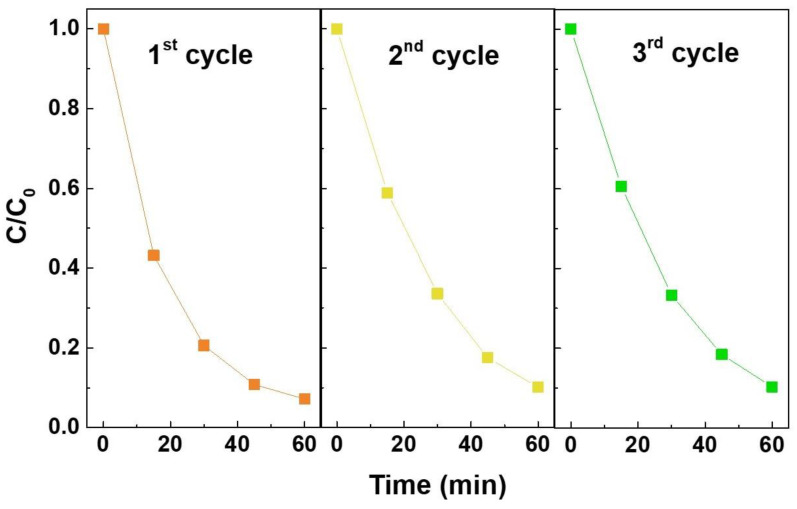
C/C_0_ comparison of the photocatalytic degradation of RhB under UV irradiation in the presence of Zn_2_SnO_4_ nanoparticles (10 min of synthesis duration). The lines are for eye guiding only.

The reusability of the ZTO nanostructures was investigated under UV light irradiation, and due to its best photocatalytic activity, the nanostructures used were the Zn_2_SnO_4_ nanoparticles obtained by a 10 min microwave-assisted hydrothermal synthesis. The C/C_0_ comparison of the photocatalytic degradation of RhB along the three reusability cycles is presented in Figure 5. As observed, after three cycles, the nanoparticles show similar performance, proving that their reusability is possible and very satisfactory, which points to the possibility of using these nanostructures for multiple water decontamination treatments. The ability to reuse these nanostructures demonstrates that they can be a part of a decontamination process that is in line with the new concept of a circular economy, which relies on maximizing the material’s lifetime and its reuse [24].

### 3.4. Photocatalytic Activity of Zn_2_SnO_4_ Nanostructures under Natural Sunlight

**Figure 6:**
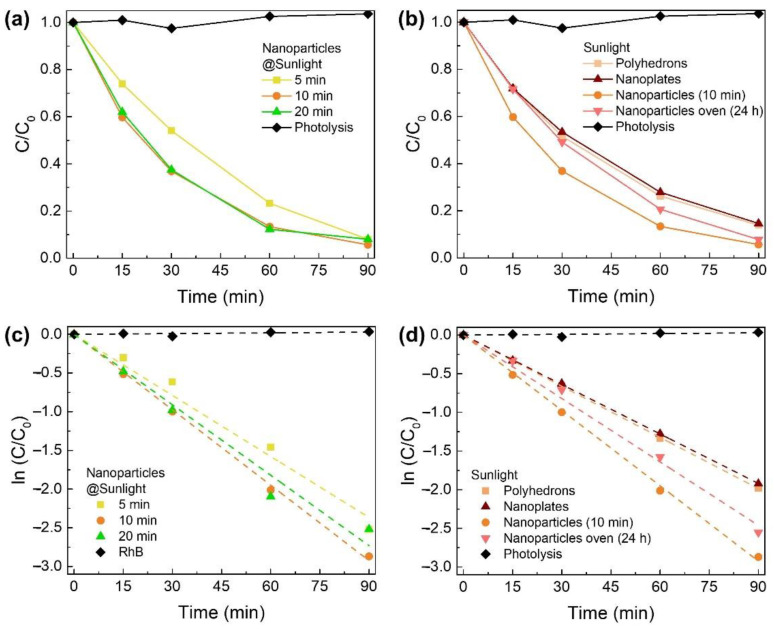
C/C_0_ comparison of the photocatalytic degradation of RhB under natural sunlight irradiation in the presence of (**a**) Zn_2_SnO_4_ nanoparticles (5, 10, and 20 min synthesis time), and (**b**) each Zn_2_SnO_4_ nanostructures (nanoplates, polyhedrons, and nanoparticles (10 min and 24 h synthesis time)). The lines in (**a**,**b**) are for eye guiding only. Kinetic parameters of the RhB degradation under natural sunlight of (**c**) Zn_2_SnO_4_ nanoparticles (5, 10, and 20 min synthesis time) and (**d**) each Zn_2_SnO_4_ nanostructure (nanoplates, polyhedrons, and nanoparticles (10 min and 24 h synthesis time)). Lines in (**c**,**d**) represent the linear fittings applied for the determination of the degradation rates. Error bars are not shown as they are not noticeable in the plot due to the low uncertainty values.

The photodegradation of RhB using the Zn_2_SnO_4_ nanostructures as catalysts under natural sunlight irradiation was also investigated. In Figure S3, the absorbance spectra of the RhB solution recorded at different degradation times under natural sunlight irradiation in the presence of each ZTO nanostructure are presented. Based on each absorbance spectrum, plots of the C/C_0_ value at each exposure time can be constructed and are presented in Figure 6a,b. Figure 6c,d presents the kinetic parameters of the RhB degradation under natural sunlight of each Zn_2_SnO_4_ nanostructure (nanoplates, polyhedrons, and nanoparticles).

Based on the kinetic parameters plots, the degradation rate of each Zn_2_SnO_4_ nanostructure was estimated, and the obtained values are presented in Table 4.

Through the analysis of the degradation rates presented in Table 4, it can be concluded that, as previously verified for the photocatalytic experiments performed under UV irradiation, among the various morphologies, the highest degradation rates were obtained for the nanoparticles. Between the various synthesis times, a 10 min duration leads to obtaining the nanoparticles with the best photocatalytic performance, with RhB degradation > 90% in just 90 min (k = 0.0325 min^−1^). Moreover, in general, the nanoparticles have a higher degradation rate (>0.0260 min^−1^) than the nanoplates (0.0213 min^−1^) and polyhedrons (0.0221 min^−1^). It is important to note that the nanoparticles obtained through a 20 min synthesis presented the smallest decrease in the degradation rate when the UV and natural sunlight are compared. As previously stated, these nanostructures present the highest E_U_, which points to the presence of a higher number of sub-gap levels that, in turn, enhance visible light response. So, as visible light represents approximately 50% of the solar spectrum, whereas UV light represents only ≈5%, it would be expected that the performance of these nanostructures would be stimulated in the tests performed under natural sunlight.

These results show high efficiency of the Zn_2_SnO_4_ nanostructures in water treatment under natural sunlight. Nevertheless, it should be considered that the obtained degradation rate values can be influenced by the injection of electrons into the conduction band of ZTO nanostructures by RhB, which can be oxidized when exposed to visible-light radiation [47]. In this scope, future work will include studies of the photocatalytic degradation of common dyes such as methyl orange and methylene blue by ZTO nanostructures when irradiated with natural sunlight. Studies using colorless compounds such as 4-chlorophenol and tetracyclines could also be interesting to perform [71].

A direct comparison of these results with what has already been reported in the literature is a challenging task since there are few works reporting the use of ZTO nanostructures as catalysts under natural sunlight, and the existing ones do not use the same organic dye as the one used in this work. Nevertheless, when compared with the literature, the presented Zn_2_SnO_4_ nanoparticles show very satisfactory performance. For example, Foletto et al. used Zn_2_SnO_4_ nanoparticles as a catalyst in the photodegradation of Reactive Red 141 dye, achieving a maximum degradation of ≈50% in 270 min under sunlight irradiation [63]. Regarding other materials, Ferreira et al. showed the application of porous ZnO nanostructures also to photodegrade RhB under sunlight, reaching a degradation rate of 0.084 min^−1^ [1]. The results obtained by these authors can be attributed to the much lower optical band gap of the ZnO nanostructures (3.25 eV) when compared with that of the Zn_2_SnO_4_ nanoparticles produced in this work (>4.18 eV) and also with their porous nature that leads to a higher surface area. However, the production of these porous nanostructures requires a calcination step at temperatures of at least 300 °C, which has a minimum duration of 2 h. This calcination step significantly increases the time required to produce the ZnO nanostructures as well as the price associated with the process.

**Figure 7:**
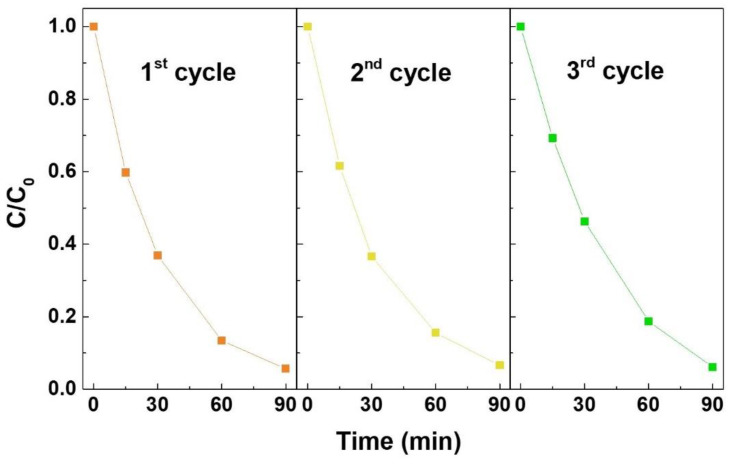
C/C_0_ comparison of the photocatalytic degradation of RhB under sunlight in the presence of Zn_2_SnO_4_ nanoparticles (10 min of synthesis duration). The lines are for eye guiding only.

As performed in the study of the RhB degradation under UV light irradiation, the reusability of the Zn_2_SnO_4_ nanoparticles (10 min) was also studied through their use in three photocatalysis cycles under natural sunlight. The C/C_0_ comparison of the photocatalytic degradation of RhB along the three reusability cycles is presented in Figure 7.

As shown in Figure 7, the nanoparticles maintain the same performance in the three cycles, showing the possibility to be reused for the degradation of RhB under natural sunlight irradiation, once again demonstrating the contribution these nanostructures can have in the new concept of a circular economy [24].

Therefore, the results herein reported clearly indicate that Zn_2_SnO_4_ nanostructures can be successfully applied for efficient RhB photodegradation under UV and sunlight irradiation. These results point to a bright future for ZTO nanostructures where Zn_2_SnO_4_ nanoparticles can be used as an eco-friendly, reusable, and efficient alternative for water decontamination.

Nevertheless, incorporating the ZTO nanostructures into a solid platform will make their applicability in large-scale water treatments more suitable and effective, thus avoiding the challenges of removing the nanomaterials (in powder form) from the water. Therefore, in the future, these ZTO nanostructures should be directly grown or impregnated in a substrate, preferentially in an eco-friendly material such as paper, which has already demonstrated great potential for photocatalytic applications and great affinity with fast microwave-assisted hydrothermal synthesis [72].

### 3.5. Photodegradation Mechanism

**Figure 8:**
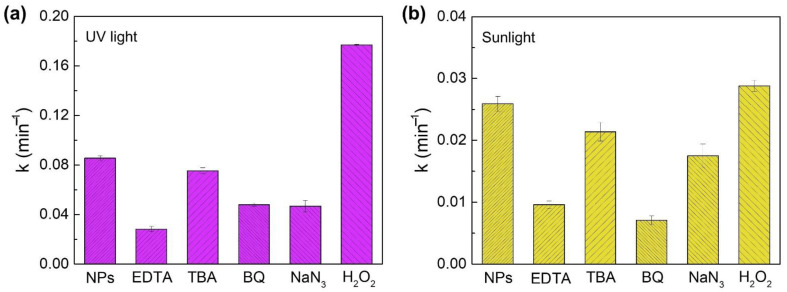
Comparison of the degradation rates of RhB under (**a**) UV light and (**b**) natural sunlight using Zn_2_SnO_4_ nanoparticles (10 min) as photocatalyst in the presence of different scavengers (EDTA, TBA, BQ, NaN_3_, and H_2_O_2_) and without adding any scavenger.

The studies performed with ROS scavengers allow for investigating the RhB photodegradation mechanism by the Zn_2_SnO_4_ nanoparticles as well as the ROS with a crucial role in it. For this study, five different scavengers were added to the RhB solution in the presence of Zn_2_SnO_4_ nanoparticles (10 min): EDTA (holes, h^+^), TBA (hydroxyl radicals, •OH), BQ (superoxide radicals, •O_2_^−^), NaN_3_ (singlets oxygen, ^1^O_2_), and H_2_O_2_ (electrons, e^−^). Similar to the previous studies, these solutions were also exposed to UV and sunlight, and the corresponding degradation rates are presented in Figure 8.

Regarding the photodegradation of RhB in the presence of the Zn_2_SnO_4_ nanoparticles under UV light irradiation, Figure 8a shows a lower degradation rate for EDTA, revealing that holes are the limiting species for the photocatalytic activity of the Zn_2_SnO_4_ nanoparticles. On the other hand, a much higher degradation rate than the one achieved without adding scavengers is observed when adding H_2_O_2_ (electrons scavenger), once again suggesting that the holes play an essential role in the photocatalytic activity of these nanostructures since the addition of this electrons scavenger prevents the recombination of the electron/hole pairs resultant from UV irradiation, which virtually creates a higher number of holes available to participate in the photocatalytic process. Nevertheless, the addition of BQ and NaN_3_ also influences the RhB degradation in the presence of the Zn_2_SnO_4_ nanoparticles, leading to a decrease in the degradation rates, showing that superoxide radicals and singlet oxygen also actively participate in the RhB degradation chemical reaction (as represented in Figure 1). These results are in agreement with our previous study [19] using Zn_2_SnO_4_ nanoparticles produced through a conventional hydrothermal route.

Figure 8b shows that under natural sunlight irradiation, similar trends are verified, with the major difference being the stronger impact of superoxide radicals on the photocatalytic process. This can be explained by the fact that with visible light, a lower concentration of electrons is available in the conduction band (only available through intrinsic defects) to promote the reaction with adsorbed oxygen to result in superoxide radicals, i.e., those radicals cannot be reestablished at a rate fast enough to assure that the degradation rate would remain the same.

These results are in accordance with what was expected since UV exposure translates into higher incident energy, capable of creating electron–hole pairs through band-to-band transitions. The experiments with ROS suggest that assuring a fast supply of holes to the system is fundamental to enhancing degradation rates. For sunlight irradiation, the photodegradation process is limited by the scavenging of superoxide radicals, as a lower concentration of electrons is available to promote the reactions required to create them.

## 4. Conclusions

While Zn_2_SnO_4_ nanostructures attract great interest for numerous applications, the synthesis times and/or the resultant yield usually do not meet the requirements, especially when considering applications that demand a high volume of material. Microwave-assisted synthesis offers a more uniform heating rate which allows for reducing the long hydrothermal synthesis times associated with the conventional route from several hours to under 60 min. Hence, this work reports the successful microwave-assisted hydrothermal syntheses at 200 °C, and without any post-annealing treatment, of three distinct Zn_2_SnO_4_ nanostructures, namely polyhedrons, nanoplates, and nanoparticles. This synthesis method allowed a reduction of the previously reported synthesis duration, achieved using a conventional oven as a heating source, from ≥24 h to 60 min for polyhedrons, 30 min for nanoplates, and 10 min for nanoparticles. The three distinct Zn_2_SnO_4_ nanostructures (polyhedrons, nanoplates, and nanoparticles) were tested as photocatalysts in the degradation of rhodamine B under both UV and natural sunlight irradiation. Among the different morphologies, the best performance was obtained for nanoparticles, in particular the ones with a 10 min synthesis duration. With these nanoparticles, RhB degradation of >90% was reached in just 60 min under UV light, and >93% in 90 min under natural sunlight. The photocatalytic mechanism of the Zn_2_SnO_4_ nanoparticles was investigated under both UV and natural sunlight, showing that under UV light, irradiation holes have a higher influence on RhB photodegradation, while under natural sunlight exposure, the superoxide radicals present a more significant effect.

Therefore, the Zn_2_SnO_4_ nanostructures produced by microwave-assisted hydrothermal synthesis at ≤200 °C and without any post-annealing treatment showed an outstanding performance in the photodegradation of an organic dye, highlighting the multifunctionality and high importance of this material.

## Figures and Tables

**Table 1:** Photocatalytic performance of different ZTO nanostructures in the degradation of pollutants.

ZTO Nanostructure	Dye	Catalyst Concentration	Radiation	Adsorption-Desorption	Photodegradation Rate (min^−1^)	Ref.
Zn_2_SnO_4_ Nanoparticles	MB (20 mg in 1 L of DW)	0.3 mg/mL	UV lamp	30 min	0.2638	[49]
Zn_2_SnO_4_ Nanocubes	MO (10 µM)	2.5 mg/mL (ethanolic solution drop-casted onto glass)	UV lamp	1 h	0.0326	[50]
	MB (10 µM)				0.0326	
Zn_2_SnO_4_ Nanoparticles	RhB (5 µM)	0.2 g/L	Visible light (λ > 420 nm, 0.5 W cm^−2^)	1 h	0.0249	[47]
	Phenol (5 µM)	0.5 mg/mL	UV (λ = 365 nm)	N.A.	N.A.	
Zn_2_SnO_4_ Nanorods	RhB (10 mg/L)	1 × 10^−5^ M	Visible light (λ ≥ 420 nm, 350 W)	60 min	0.00264	[48]
	MO (10 mg/L)				0.00141	
Zn_2_SnO_4_ Nanoparticles	RhB (5 mg/L)	0.8 mg/mL	Visible light (100 mW·cm^−2^)	30 min	10.1 × 10^−4^	[19]
			UV (λ = 254 nm, 35 W/m^2^)		0.0340	
	MB (1.5 mg/L)	0.8 mg/mL	UV (λ = 254 nm, 35 W/m^2^)	30 min	0.0273	
Zn_2_SnO_4_ Polyhedrons	RhB (5 mg/L)	0.8 mg/mL	Visible light (100 mW·cm^−2^)	30 min	4.7 × 10^−4^	[19]
			UV (λ = 254 nm, 35 W/m^2^)		0.0132	
	MB (1.5 mg/L)	0.8 mg/mL	UV (λ = 254 nm, 35 W/m^2^)	30 min	0.0164	
ZnSnO_3_ Nanowires	RhB (5 mg/L)	0.8 mg/mL	Visible light (100 mW·cm^−2^)	30 min	8.1 × 10^−4^	[19]
			UV (λ = 254 nm, 35 W/m^2^)		0.0330	
	MB (1.5 mg/L)	0.8 mg/mL	UV (λ = 254 nm, 35 W/m^2^)	30 min	0.0381	

**Table 2:** Size, optical band gap, and Urbach energy of Zn_2_SnO_4_ nanoparticles (different synthesis duration), polyhedrons, and nanoplates. The diameter values were determined through the ImageJ software. The presented values correspond to the average of 10 measurements.

Sample	Diameter (nm)	Optical Band Gap (eV)	Urbach Energy, E_U_ (eV)
Nanoparticles (5 min)	23 ± 2	4.25	0.07
Nanoparticles (10 min)	31 ± 4	4.18	0.11
Nanoparticles (20 min)	28 ± 3	4.20	0.18
Nanoparticles (24 h @oven)	18 ± 3 ^1^	3.95 ^1^	0.17 ^1^
Polyhedrons (60 min)	313 ± 99	3.98	0.06
Nanoplates (30 min)	244 ± 27	3.84	0.07

^1^ Values from reference [19].

**Table 3:** Degradation rates of RhB in the presence of each ZTO nanostructures under UV light.

Sample	Degradation Rate (min^−1^)
Nanoparticles (5 min)	0.0374 ± 0.0001
Nanoparticles (10 min)	0.0471 ± 0.0002
Nanoparticles (20 min)	0.0366 ± 0.0005
Nanoparticles (24 h @oven)	0.0340 ± 0.0016 ^1^
Polyhedrons (60 min)	0.0151 ± 0.0002
Nanoplates (30 min)	0.0297 ± 0.0006

^1^ Value from reference [19].

**Table 4:** Degradation rates of RhB in the presence of each ZTO nanostructures under sunlight.

Sample	Degradation Rate (min^−1^)
Nanoparticles (5 min)	0.0263 ± 0.0012
Nanoparticles (10 min)	0.0325 ± 0.0004
Nanoparticles (20 min)	0.0304 ± 0.0016
Nanoparticles (24 h@oven)	0.0273 ± 0.0007
Polyhedrons (60 min)	0.0221 ± 0.0001
Nanoplates (30 min)	0.0213 ± 0.0001

## Data Availability

The data presented in this study are available on request from the corresponding author.

## Supplementary Materials

The following supporting information can be downloaded at: https://www.mdpi.com/article/10.3390/nano12122119/s1, Figure S1: Diffuse reflectance and Kubelka–Munk plots of ZTO nanostructures, Figure S2: Absorbance spectra of RhB degradation under UV light, Figure S3: Absorbance spectra of RhB degradation under natural sunlight.
