# Supplementary material for: Microwave-Assisted Synthesis of Zn2SnO4 Nanostructures for Photodegradation of Rhodamine B under UV and Sunlight
Source: Nanomaterials (Basel). 2022 Jun 20;12(12):2119. doi: 10.3390/nano12122119 (PMC9231267; doi:10.3390/nano12122119)
Supplement: Supplementary file 1 [file nanomaterials-12-02119-s001.zip › nanomaterials-1778289-supplementary conversion.pdf]

nanomaterials

Supporting Information

Microwave-Assisted Synthesis of Zn_2SnO_4 Nanostructures for Photodegradation of Rhodamine B under UV and Sunlight

Ana Rovisco *,†, Maria Morais †, Rita Branquinho, Elvira Fortunato, Rodrigo Martins and Pedro Barquinha *

CENIMAT/i3N, Department of Materials Science, NOVA School of Science and Technology (FCT-NOVA) and CEMOP/UNINOVA, NOVA University Lisbon, Campus de Caparica, 2829-516 Caparica, Portugal; md.morais@campus.fct.unl.pt (M.M.); ritasba@fct.unl.pt (R.B.); emf@fct.unl.pt (E.F.); rfpm@fct.unl.pt (R.M.)

* Correspondence: a.rovisco@fct.unl.pt (A.R.); pmcb@fct.unl.pt (P.B.)

† These authors contributed equally to this work.

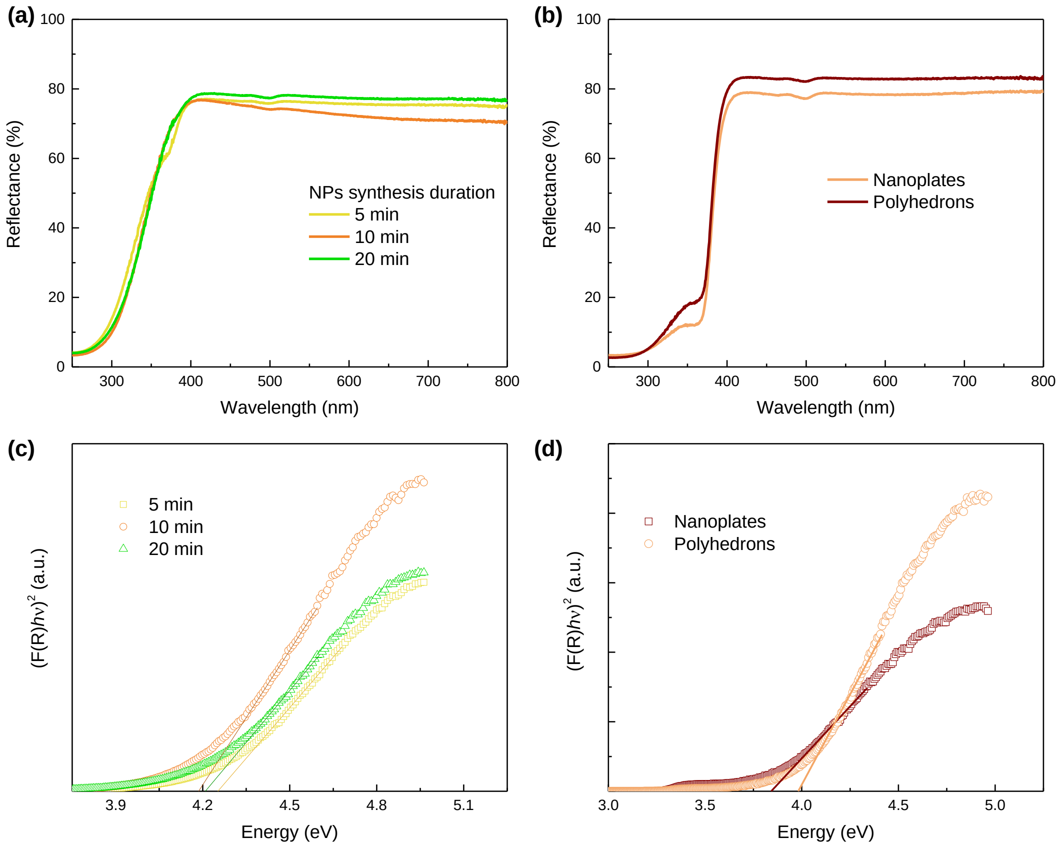

Figure S1. (**a** and **b**) Diffuse reflectance spectra and (**c** and **d**) Kulbelka-Munk function of Zn_2SnO_4 nanoparticles, and nanoplates and polyhedrons, respectively. The lines in (**c**) and (**d**) represent the linear fittings applied for the determination of the optical band gap values.

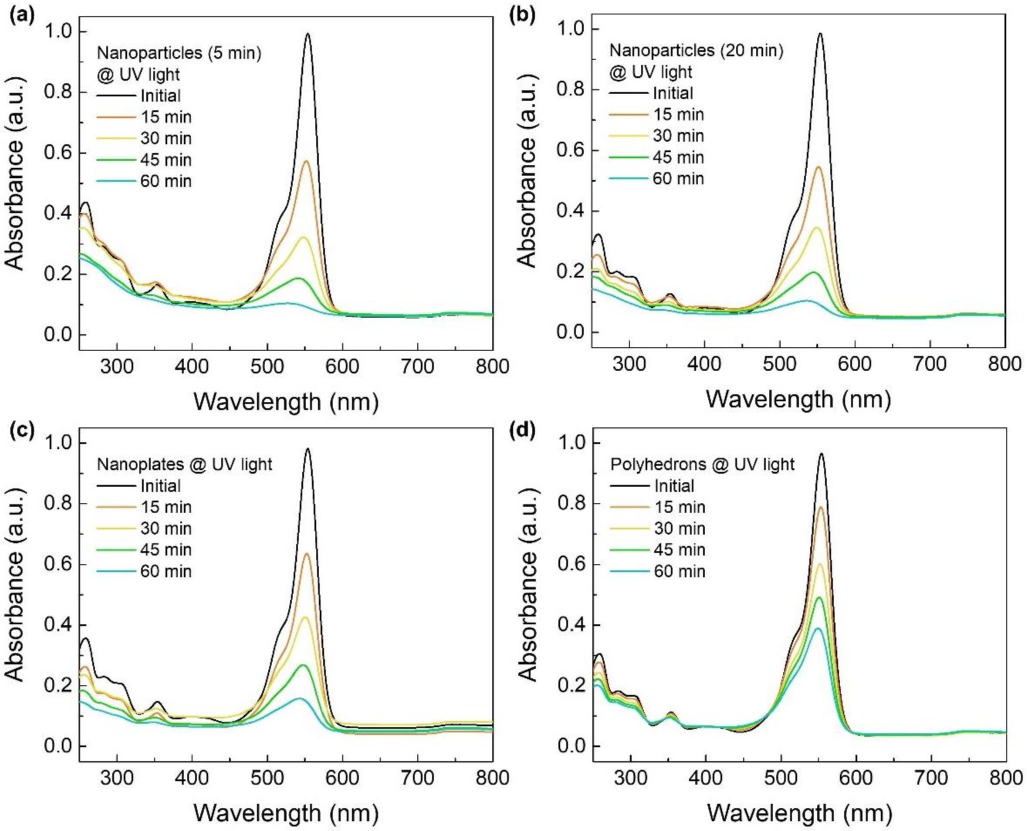

Figure S2. Absorbance spectra of the RhB degradation under UV light irradiation in the presence of Zn_2SnO_4 (**a**) nanoparticles (5 min synthesis time), (**b**) nanoparticles (20 min synthesis time), (**c**) nanoplates, and (**d**) polyhedrons.

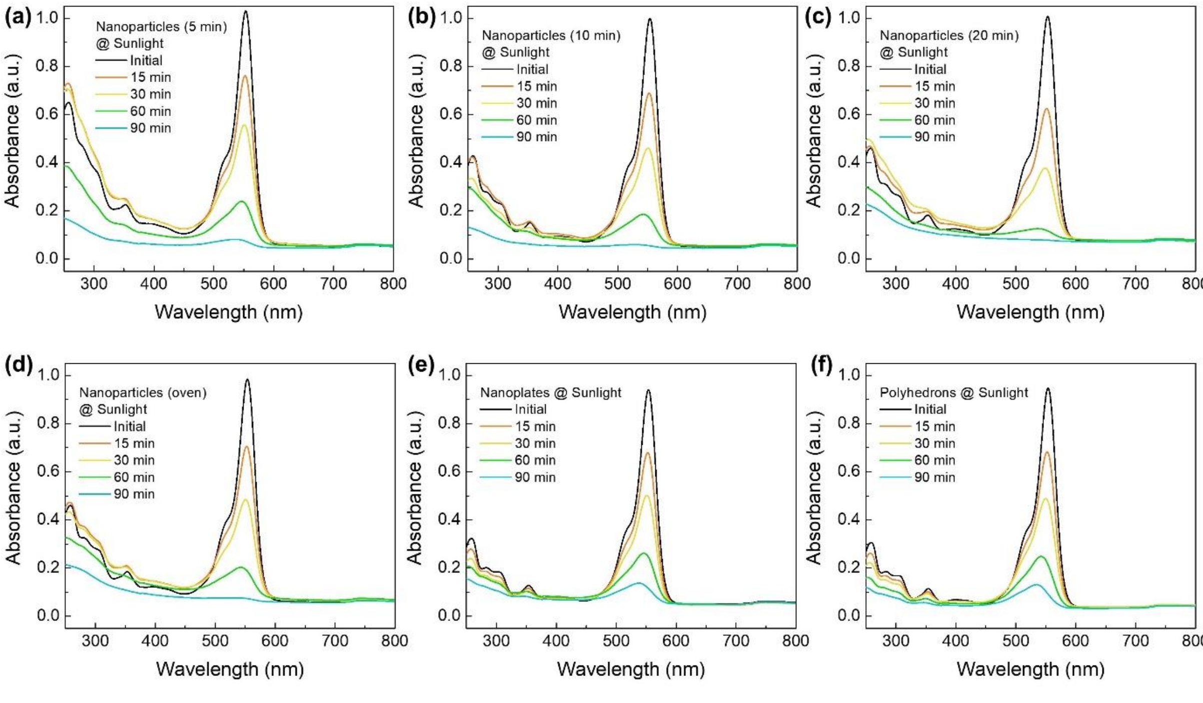

Figure S3. Absorbance spectra of the RhB degradation under natural sunlight irradiation in the presence of Zn_2SnO_4 (**a**) nanoparticles (5 min synthesis time), (**b**) nanoparticles (10 min synthesis time), (**c**) nanoparticles (20 min synthesis time), (**d**) nanoparticles (conventional oven), (**e**) nanoplates, and (**f**) polyhedrons.
